# Evolution Characteristics and Formation Mechanism of Production-Living-Ecological Space in China: Perspective of Main Function Zones

**DOI:** 10.3390/ijerph19169910

**Published:** 2022-08-11

**Authors:** Ning Xu, Wanxu Chen, Sipei Pan, Jiale Liang, Jiaojiao Bian

**Affiliations:** 1 School of Political Science and Public Administration, Henan Normal University, Xinxiang 453007, China; 2 School of Geography and Information Engineering, China University of Geosciences, Wuhan 430078, China; 3 College of Public Administration, Nanjing Agricultural University, Nanjing 210095, China; 4 School of Geography and Oceanography Sciences, Nanjing University, Nanjing 210023, China

**Keywords:** main function zone, territorial space, production-living-ecological space, influencing factors, formation mechanism, China

## Abstract

The main function zone (MFZ) is the major strategy of China’s economic development and ecological environment protection. Clarifying the logical relationship between “MFZ strategy” and “territorial spatial layout” is vital to construct regional economic layout and territorial spatial supporting system of high-quality development. However, few studies have revealed the evolution process and formation mechanism of the production-living-ecological space (PLES) structure of China’s MFZ over a long period of time. To bridge the gap, based on the land use dataset in China from 1980 to 2020, this study analyzed the evolution patterns of PLES in China’s MFZs using multiple methods and measured the formation mechanism of PLES in different types of MFZs with the GeoDetector model. Results showed that the spatial structure of China’s national territory has evolved drastically in the past 40 years, showing significant horizontal regional differentiation and vertical gradient differentiation. Ecological space has been continuously decreasing, while production space and living space have been continuously increasing, and the evolution of PLES varied significantly in different MFZs. During the study period, the gravity center of PLES in China all moved westward. The spatial distribution pattern of production space and living space was from northeast to southwest, and the ecological space was from east to west. The evolution of China’s territorial spatial structure was subject to the combined effects of natural and socio-economic factors, exhibiting significant differences in different MFZs. Land use intensity had the most prominent influence on the formation of PLES, followed by elevation. The influences of different factors on PLES structure were strengthened mainly through two types of nonlinear enhancement and dual-factor enhancement. This study can provide scientific support for the optimal management and high-quality development of territorial space in China.

## 1. Introduction

The past 40 years has witnessed remarkable achievements in China’s socio-economic development and has also brought about drastic changes in territorial spatial pattern [1,2]. The long-standing lack of spatial layout planning in China has led to disordered territorial spatial development, tightening resource constraints, and imbalanced regional development issues in the process of rapid urbanization in China [3,4,5]. In 2021, the *Outline of the People’s Republic of China 14th Five-Year Plan for National Economic and Social Development and Long-Range Objectives for 2035* put forward the further implementation of regional major strategy, regional coordinated development strategy, main function zone (MFZ) strategy to improve the system of regional harmonious development mechanism, and achieve high-quality development of the regional economic layout and the supporting system for the territorial spatial development [6]. How to build high-quality development of regional economic layout and territorial spatial support system has become the current focus of attention [5,7]. The planning of MFZ is a major innovation of coordinated regional development in China. It is an innovative spatial control method proposed to solve the problems of disordered territorial spatial development and imbalanced regional development under China’s rapid economic growth [8,9]. The MFZ was first proposed in China’s 11th Five-Year Plan [8]. Since then, MFZ has evolved from planning to regional strategy to national basic system and has currently become the overall plan of China’s “one blueprint to the end” [9,10]. According to the *National Plan for Main Function Zones* issued by the State Council and the *Plan for China’s Main function Zoning* (*V1.0*), the MFZs are divided into optimized development zone, key development zone, restricted development zone, and prohibited development zone according to their development modes [11,12]. The restricted development zones are divided into main agricultural production zone and key eco-function zone. As prohibited development zone is a kind of functional zone superimposed on the other three functional zones, the area of which is relatively small compared with the other three functional zones, it is not considered in this study [13].

Pieces of previous research have been conducted on the MFZ, mainly focusing on the conceptual theory [10,14,15,16], zoning [17], structural analysis [15], monitoring and evaluation [5,18], simulation [19,20,21], coordinated development [5,7,22], pattern optimization [23,24,25,26], influence mechanism [5,27], and supporting policies [28]. As the strategic background of national planning, the MFZ is the prospect of the overall pattern of China’s territorial spatial protection and development in the future [11,14], which can guide the quantity distribution and spatial layout of production-living-ecological space (PLES) through territorial spatial planning, three-zones and three-lines management and control [11]. However, previous literature on the evolution of the spatial structure of the MFZ mainly analyzed its structural evolution based on socio-economic development (e.g., per capita GDP, population, urbanization) [29,30], ecological function [31], and construction land [13,32]. Few studies have revealed the evolution process and formation mechanism of the PLES structure of China’s MFZ over a long period of time. Thus, a systematical review of the evolution of China’s PLES structure over a long period of time is an important basis for exploring the optimization strategy of the pattern of MFZ and improving the development strategy and spatial governance system of China’s territorial space [33].

PLES is the carrier and path of territorial spatial optimization, which can not only reflect the development and utilization orientation of national strategy at the level of territorial space but also mirror the public’s real demands for PLES [16]. Land is the carrier of ecological protection and high-quality development, and the coordinated development of PLES will promote such protection and development [5]. The geographical space classification system of PLES is a comprehensive land spatial zoning method and the related research is mainly focused on China [34]. In China, the classification of PLES is based on the theory of multi-functionality of land use in Europe [35]. Thus, land use change is a direct reflection of PLES change. Previous studies on PLES mainly focused on PLES theory [16], classification [36], pattern evolution [17,37,38], optimization coordination, and conflict regulation [39,40], whereas few studies explored the structural evolution characteristics of PLES based on MFZ. PLES inherits the strategic positioning in territorial spatial planning under the MFZ strategy and is reflected in the quantity and spatial layout of PLES [41]. MFZs differ significantly in economic development, development intensity, resource and environmental carrying capacity, development potential, and development direction. Therefore, it is particularly necessary to understand the driving mechanism of the MFZ structure of different types of MFZs, which will help to promote the formation of a spatial development pattern with effective main function constraints and orderly territorial space development. Previous studies have explored the driving mechanism of natural factors and socio-economic conditions on territorial spatial differentiation in river basins and mountainous areas [4,41], but research on the process of PLES change and regional differentiation mechanism of national MFZ is still insufficient.

China is a vast country with significant regional differences in natural environment, resource endowment, stage, and characteristics of socio-economic development. It is vital to explore the evolution process of PLES structure and regional differentiation mechanism of China’s MFZ over a long period of time for the construction of high-quality regional economic layout and territorial spatial support system [42]. Therefore, based on the land use data in China in 1980, 1990, 2000, 2010, and 2020, this study introduced land spatial transfer matrix, landscape pattern metrics, and standard deviation ellipse to measure the evolution characteristics of PLES structure of China’s MFZ. Meanwhile, with the help of GeoDetector model, the formation mechanism of the regional differentiation of PLES in four types of MFZs is explored. Specifically, the study aims to (1) Identify the spatio-temporal evolution patterns of PLES in China from the perspective of MFZ. (2) Explore the formation mechanism of PLES in China. (3) Provide theoretical reference and decision-making basis for the development of national space and the optimization of MFZ.

## 2. Data Sources and Methods

### 2.1. Data Sources

**Figure 1:**
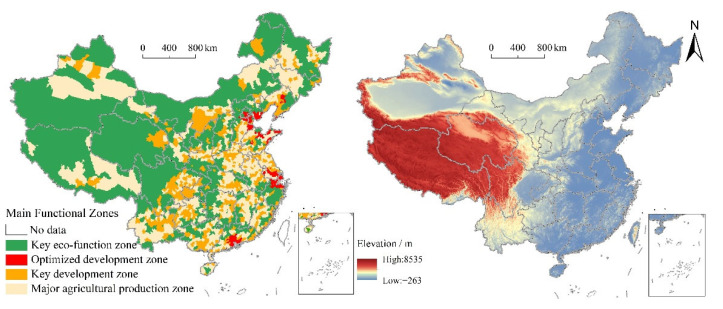
Spatial distribution of MFZ and elevation in China.

The data of 1 × 1 km land use data in China in 1980, 1990, 2000, 2010, and 2020 were obtained from Resources and Environmental Science and Data Center of Chinese Academy of Sciences (RESDC) (http://www.resdc.cn/, accessed on 12 July 2022). Based on Landsat TM/ETM and Landsat 8 remote sensing images, this dataset was generated by Liu et al. through manual visual interpretation with 5-year interval [43]. Land use types include 6 first-level types and 25 second-level types. A distance of 1 km resolution DEM, annual precipitation, and annual mean temperature data were also obtained from RESDC. The 2000, 2010, and 2020 collections of China’s population density data are supplied by the WorldPop website (https://www.worldpop.org/, accessed on 12 July 2022), with a resolution of 100 × 100 m. The data of MFZ used in this study were derived from the National Planning for MFZ issued by The State Council and the *Plan for China’s Main Function Zoning* (*V1.0*) [11]. To fully reveal the evolution process and formation mechanism of PLES in different MFZs, this study combined optimized development zone, key development zone, major agricultural production zone, and key eco-function zone (Figure 1).

### 2.2. Classification System of Production-Living-Ecological Space

Building a scientific and reasonable classification system of PLES is the premise and basis for studying the structural evolution of PLES [44]. Territorial spatial pattern is a comprehensive reflection of the interaction and coupling between natural ecological process and humanistic social system [45], and territorial space is a multi-functional complex [18,34,46,47]. Scholars have conducted a large number of studies on the classification of PLES, mainly based on the dominant functions of different land use types [5,44]. Based on previous studies, this study took the multi-function of territorial space as the entry point, combined with the land use classification system of Chinese Academy of Sciences and *Current Land Use Classification* (GB/T21010-2007), classified the PLES in China, and constructed the classification system of PLES in China for specific reference [5,48,49,50] (Table 1).

### 2.3. Methods

#### 2.3.1. National Spatial Transfer Matrix

The national spatial transfer matrix takes the land use transfer area as the matrix, reflecting the structure and current situation of the dynamic change of land use [51]. Transfer matrix is usually used to analyze and estimate the rate of land use change and quantitatively describe the structural characteristics of land use [49]. The specific equation is as follow:

(1) $$S_{ij}=\left|\begin{array}{cccc} s_{11} & s_{12} & \cdot \cdot \cdot & s_{1n}\\ s_{21} & s_{22} & \cdot \cdot \cdot & s_{2n}\\ \begin{array}{l} s_{31}\\ \cdot \cdot \cdot \end{array} & \begin{array}{l} s_{32}\\ \cdot \cdot \cdot \end{array} & \begin{array}{l} \cdot \cdot \cdot \\ \cdot \cdot \cdot \end{array} & \begin{array}{l} s_{3n}\\ \cdot \cdot \cdot \end{array}\\ s_{n1} & s_{n2} & \cdot \cdot \cdot & s_{nn} \end{array}\right|$$

where, *S_ij_* is the area of category *i* territorial spatial type at the early stage of the study converted to category *j* territorial spatial type at the late stage of the study; *n* is the number of types of territorial spatial utilization.

#### 2.3.2. Landscape Pattern Metrics

Landscape pattern metrics can well represent landscape dynamics and functions [52,53]. The evolution patterns of territorial space in spatial form are subject to different aspects of landscape pattern, such as the area, density, and proximity. Meanwhile, landscape structure, function, and change are scale dependent [54]. Thus, scale effects must be incorporated when selecting specific indicators to characterize different aspects. With reference to relevant studies [55,56,57,58] and the actual situation of the study scale of this research, five landscape pattern indices, namely percentage of landscape (PLAND), patch cohesion index (COHESION), patch density (PD), largest patch index (LPI), and mean Euclidean nearest neighbor distance (ENN_MN), were selected from the aspects of proximity, area edge, and aggregation dispersion to measure the evolution process of landscape patterns in the recent 40 years in China.

#### 2.3.3. Standard Deviation Ellipse

Standard deviation ellipse method can quantitatively and accurately reveal the spatial distribution characteristics of geographical and socio-economic elements, such as centrality, spatial range, and evolution direction, through the parameters of ellipse center, long axis, short axis, azimuth angle, and flattening [59,60]. In this study, standard deviation ellipse was used to identify the gravity center position and its spatial movement trend of territorial spatial type area. The specific equations are as follows:

(2) $${\bar{X}}_{w}=\sum_{i=1}^{n}w_{i}x_{i}/\sum_{i=1}^{n}w_{i}$$

(3) $${\bar{Y}}_{w}=\sum_{i=1}^{n}w_{i}y_{i}/\sum_{i=1}^{n}w_{i}$$

(4) $$\theta =\arctan\left[\left(\sum_{i=1}^{n}x_{i}^{\prime 2}-\sum_{i=1}^{n}y_{i}^{\prime 2}\right)+\sqrt{{\left(\sum_{i=1}^{n}x_{i}^{\prime 2}-\sum_{i=1}^{n}y_{i}^{\prime 2}\right)}^{2}+4{\left(\sum_{i=1}^{n}x_{i}^{\prime }y_{i}^{\prime }\right)}^{2}}\right]/2\sum_{i=1}^{n}x_{i}^{\prime }y_{i}^{\prime }$$

(5) $$\delta_{x}=\sqrt{\sum_{i=1}^{n}{\left(x_{i}^{'}\cos\theta -y_{i}^{'}\sin\theta \right)}^{2}/n},\delta_{y}=\sqrt{\sum_{i=1}^{n}{\left(x_{i}^{'}\sin\theta -y_{i}^{'}\cos\theta \right)}^{2}/n}$$

where $\left({\bar{X}}_{w},{\bar{Y}}_{w}\right)$ is the weighted average center; (*x_i_*, *y_i_*) is the geometric center coordinate of county unit *i*; *w_i_* is the weight; *θ* is azimuth; *δx* and *δy* are, respectively, the standard deviation along the major axis and the minor axis.

#### 2.3.4. GeoDetector Model

To reveal the evolution mechanism of PLES structure in different MFZs in China, this study intended to use the Geodetector model to quantitatively detect the regional differentiation of PLES and its driving forces [61,62]. This method can effectively detect the influence of various factors and identify the strength of interaction among multiple factors [63]. The specific equation is as follow:

(6) $$P_{D,H}=1-\frac{1}{N\sigma^{2}}\sum_{h=1}^{L}N_{h}\sigma_{h}^{2}$$

where *P_D,H_* is the detection power indicator of factors influencing the differentiation of PLES, and it values 0~1. If the independent variable has stronger explanatory power to the dependent variable, the value of *P_D,H_* will be higher. *N* and *N_h_* are the number of sample units in the study area and the number of sample units in the sub-level area, respectively. *L* is the number of layers or partitions of independent variables or dependent variables; *σ*^2^ and *σ_h_*^2^ are the variances of the whole region and the sub-region. GeoDetector mainly includes four detectors, namely factor detection, interaction detection, risk area detection, and ecological detection. This study focused on the formation mechanism of PLES detection, so factor detection and interaction detection are selected for quantitative elaboration and analysis. The types of interaction between two factors can be divided into the following five categories (Table 2).

Referring to previous studies, territorial spatial evolution is formed under the comprehensive action of natural and socio-economic factors [4,49,64]. In this study, six influencing factors, including land use intensity (*X*_1_), normalized difference vegetation index (NDVI, *X*_2_), population density (*X*_3_), annual average temperature (*X_4_*), annual average precipitation (*X*_5_), and average elevation (*X*_6_) were selected from two aspects of natural factors and socio-economic factors. Socio-economic factors mainly include *X*_1_ and *X*_3_, among which X_1_ can effectively measure the intensity of human activities [65]. *X*_3_ is used to represent the pressure of population pressure on territorial space development and utilization [66]. *X*_2_ was used to characterize the effect of vegetation growth on PLES structure. *X*_4_ and *X*_5_ are used to represent the influence of climate factors on the evolution of territorial spatial structure, while *X*_6_ is used to represent the impact of topographic factors on territorial spatial evolution [4,49,64]. The PLES and influencing factors in 2000, 2010, and 2020 were spatialized by ArcGIS 10.3 software, and the PLES and influencing factors index database of different MFZs of county units in China was constructed.

## 3. Results

### 3.1. Spatio-Temporal Evolution Pattern of Territorial Space in China from 1980 to 2020

**Figure 2:**
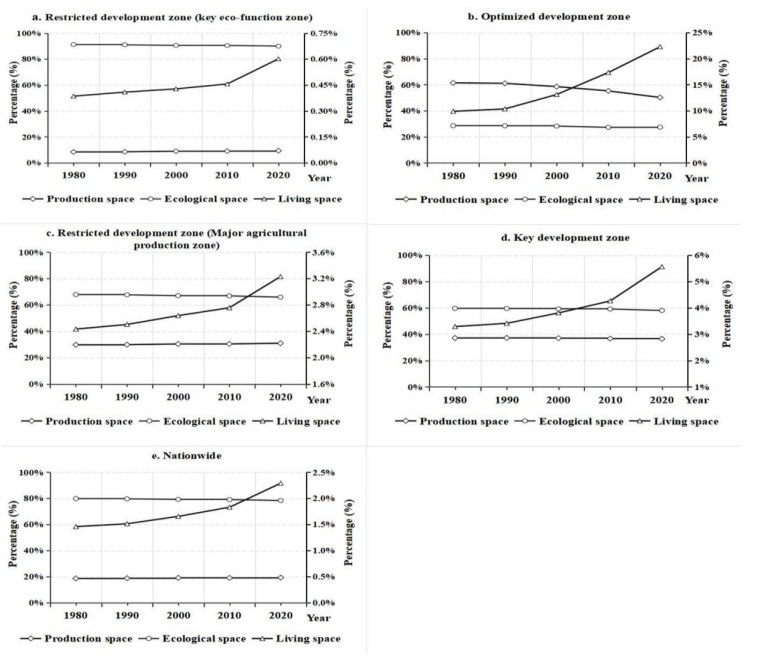
Proportion of PLES in major functional zones in China (%). Note: Production space and ecological space correspond to the main axis (**left**); living space corresponds to the sub-axis (**right**).

From 1980 to 2020, ecological space played an absolutely dominant role in China’s territorial space, accounting for a significantly higher proportion than production space and living space. During the study period, the proportion of ecological space continued to decrease from 79.87% in 1980 to 78.40% in 2020. Production space and living space continued to increase, with production space increasing from 18.67% in 1980 to 19.30% in 2020 and living space from 1.46% in 1980 to 2.29% in 2020 (Figure 2). There are significant differences in the proportion of PLES in different types of MFZs. Specifically, ecological space of different types of MFZs showed a reduction in the overall trend. Among them, the ecological space area of restricted development zone (key eco-function zone) accounts for the highest proportion (nearly 90%), followed by restricted development zone (major agricultural production zone), accounting for about 66%, and the ecological space of optimized development zone accounts for the lowest proportion, less than 30%.

During the study period, the production space of different types of MFZs varied greatly. The proportion of production space in optimized development zone was the highest (>50%) and showed a gradual decline trend, followed by the proportion of production space in key development zone (>36%), which also showed a continuous decline trend. The proportion of production space in restricted development zone (key eco-function zone) is <10%, and the proportion of production space in restricted development zone (major agricultural production zone) is approximately 30%, both showing a continuous increase trend. During the study period, the living space of different types of MFZs showed an overall increasing trend. The living space proportion of restricted development zone (key eco-function zone) was the lowest (<0.60%), followed by restricted development zone (major agricultural production zone), which accounted for <4%; while the living space proportion of optimized development zone was the highest, which increased from 9.90% in 1980 to 22.28% in 2020.

**Figure 3:**
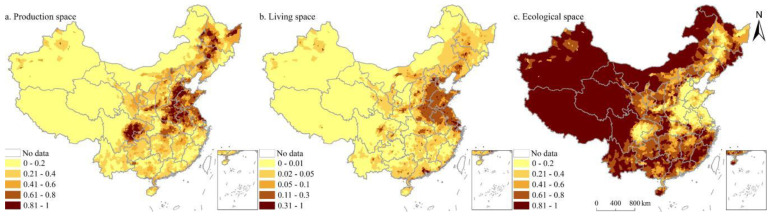
Spatial distribution of PLES at county level in China in 2020.

**Figure 4:**
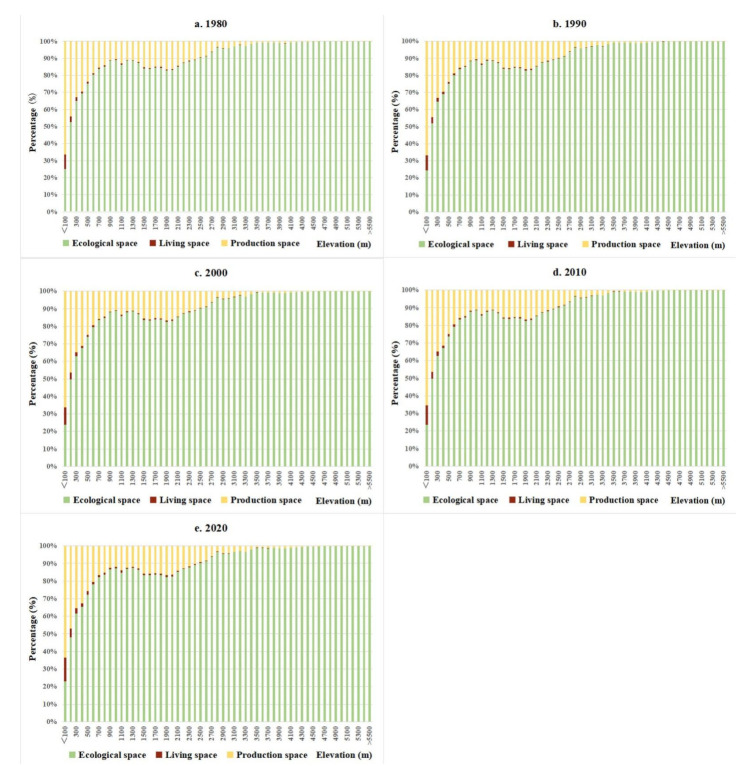
Changes in the proportion of PLES in different elevations in China from 1980–2020.

**Figure 5:**
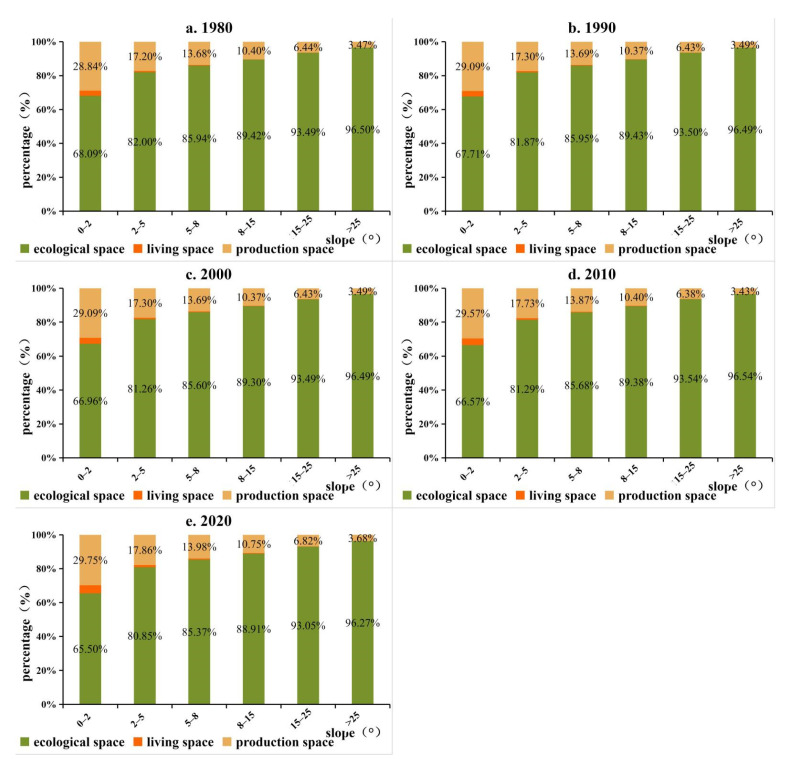
Changes in the proportion of PLES in different slopes in China from 1980–2020.

Owing to its vast territory, complex terrain, and diverse climate, China’s territorial space utilization types are significantly different. Production space and living space showed similar spatial distribution patterns during the study period, mainly distributed in the east of Hu line (Figure 3). Specifically, production space and living space are mainly distributed in the Sichuan Basin, North China Plain, Guanzhong Plain, Northeast Plain, the Middle-Lower Yangtze River Plain, and the Pearl River Delta Region. In addition, there is more production space and living space in the surrounding areas of provincial capital cities, urban agglomeration areas, and major transportation routes. Ecological space is concentrated in the west of Hu line and mountainous areas in the east of Hu line, such as the Lesser Khingan Mountains, Changbai Mountains, T’ai-hang Mountains, Dabie Mountains, Wushan Mountains, Xuefeng Mountains, Nan Mountains, and Wuyi Mountains. Besides, the vertical gradient of China’s territorial space is obviously differentiated (Figure 4). A total of 27.60% of the total territorial space is concentrated below 500 m, accounting for 15.81% within 500~1000 m, 17.88% within 1000~1500 m, and 6.68% within 1500~2000 m. The proportion of territorial space above 4000 m is 20.00%. From 1980 to 2020, the proportion of PLES at different elevations did not change significantly. With the increase in elevation, the proportion of living space continued to decrease, the proportion of production space first decreased, then increased, and then decreased, whereas the proportion of ecological space first increased, then decreased, and then increased. Specifically, the proportion of production space in the range of 0~1200 m continued to decrease and gradually increased in the range of 1200~1600 m, and then showed an overall decreasing trend. The change trend of ecological space showed the opposite. Below 100 m, production space was more than 50%, while ecological space was over 30%. The ecological space between 100 and 200 m accounted for >50%, while the production space accounted for >40%. In China, the proportions of 0~2°, 2~5°, 5~8°, 8~15°, 15~25°, and >25° are 40.89%, 16.95%, 10.51%, 15.77%, 10.88%, and 5.01%, respectively. It can be found that the territorial space is mainly concentrated below 5° (Figure 5). From 1980 to 2020, the proportion of PLES in different slopes had little change. The proportion of production space and living space decreased with the increase in slope, while the proportion of ecological space increased.

### 3.2. Spatial Transfer Matrix of Production-Living-Ecological Space in China from 1980 to 2020

**Figure 6:**
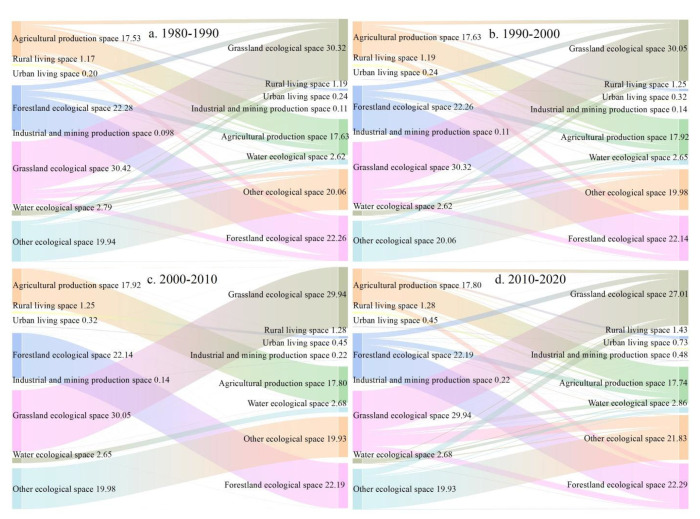
Sankey diagram of territorial space transfer matrix (Unit: 10^5^ km^2^).

Based on the conversion of PLES between 1980 and 2020, this study visualized land use transition matrices of four periods by using Sankey diagram (Figure 6). From 1980 to 1990, the range of grassland ecological space and agricultural production space to forestland ecological space was 25,325.47 km^2^ and 251,574.11 km^2^, respectively. The area of grassland ecological space converted to other space was the largest, reaching 681,424.70 km^2^. Meanwhile, the area of other space converted to grassland ecological space was also the largest, reaching 672,171.73 km^2^. The area of agricultural production space converted to other space reached 578,143.31 km^2^. The conversion of industrial and mining production space to other space was the smallest, and the area of other space to forestland ecological space was also relatively large, reaching 543,864.98 km^2^. From 1990 to 2000, the conversion of forestland ecological space to agricultural production space, forestland ecological space to grassland ecological space, and other ecological space to grassland ecological space were relatively evident, accounting for 21.95%, 21.34%, and 18.19% of the national spatial transformation area, respectively. From 1990 to 2000, the area of grassland ecological space converted to other space was the largest, reaching 695,586.91 km^2^, followed by agricultural production space and forestland production space converted to other space, reaching 582,231.59 km^2^ and 555,507.73 km^2^. The grassland ecological space converted from other space was the largest, reaching 668,095.14 km^2^, followed by the agricultural production space and forestland ecological space converted from other space, reaching 611,079.11 km^2^ and 543,198.54 km^2^, respectively.

From 2000 to 2010, grassland ecological space turned into agricultural production space, agricultural production space turned into urban living space, and grassland ecological space turned into forestland ecological space, accounting for 11.94%, 9.51%, and 7.94% of the transformation area of territorial space from 2000 to 2010, respectively. From 2010 to 2020, the transition between other ecological space and grassland ecological space was the most intense, accounting for 28.73% of the national spatial transformation area from 2010 to 2020, followed by grassland ecological space to forestland ecological space, accounting for 9.73% of the national spatial transformation area. From 2010 to 2020, the area of grassland ecological space converted to other space was 1,066,650.67 km^2^, followed by the agricultural production space converted to other space, reaching 607,092.58 km^2^. From 2010 to 2020, the area of other space converted to grassland ecological space was the largest, reaching 7,760,669.25 km^2^, followed by other space to other ecological space and agricultural production space, 615,643.26 km^2^ and 603,473.00 km^2^, respectively.

### 3.3. Landscape Pattern of Production-Living-Ecological Space in China from 1980 to 2020

**Figure 7:**
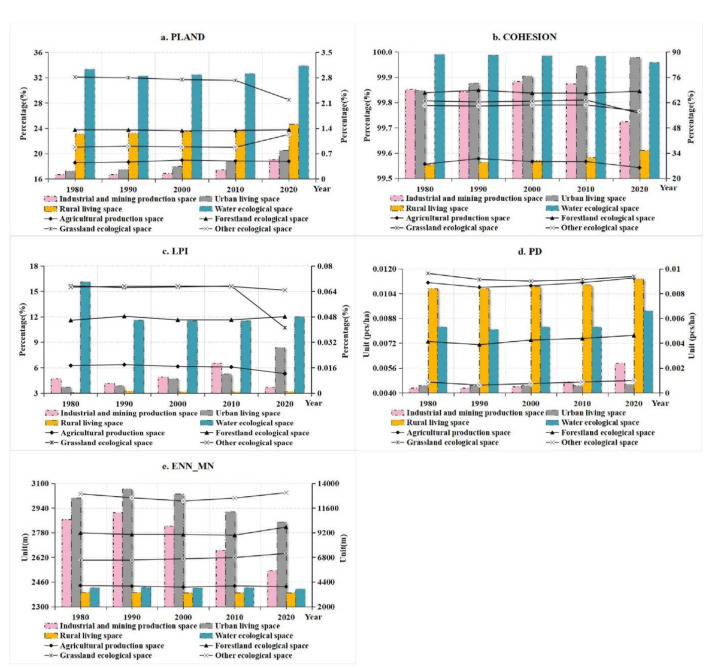
Landscape pattern index of territorial space in China during 1980–2020.

During the study period, the proportions of industrial and mining production space, urban living space, and rural living space continued to increase, while the water ecological space decreased from 1980 to 1990 and continued to increase from 1990 to 2020. From 1980 to 2000, the agricultural production space increased continuously, and then decreased continuously in the following 20 years. On the contrary, the forestland ecological space decreased continuously from 1980 to 2000 and increased continuously in the following 20 years. During the study period, grassland ecological space continued to decrease, while other ecological space showed a general decline trend. The COHESION index of ecological space was significantly higher than that of living space and production space, and the COHESION index of rural living space was the lowest (Figure 7b). During the study period, the COHESION index of urban living space and rural living space continued to increase, indicating that the agglomeration degree of living space increased significantly, while the ecological space of water area continued to decrease, and the COHESION index of industrial and mining production space increased first and then decreased, while other types of territorial space had little change. The LPI of forestland ecological space, grassland ecological space, and other ecological space was significantly higher than that of other territorial space types, and the proportion of industrial and mining production space, urban living space, and rural living space was relatively low (Figure 7c). The PD index of agricultural production space and grassland ecological space was higher than that of industrial and mining production space and urban living space (Figure 7d). The PD of industrial and mining production space, urban living space, rural living space, and forestland ecological space increased during the study period. The ENN_MN of industrial and mining production space and urban living space is relatively larger, followed by rural living space and water ecological space (Figure 7e).

### 3.4. Changes in the Direction of Territorial Expansion in China from 1980 to 2020

**Figure 8:**
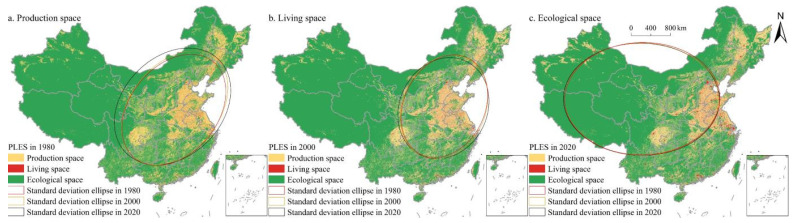
Standard deviation ellipse of PLES pattern in China during 1980–2020.

Based on Equations (2)–(5), this study drew the standard deviation ellipse of PLES in China from 1980 to 2020, which was used to analyze the overall patterns of China’s territorial spatial distribution and its spatial movement direction (Figure 8). Production space and living space form a northeast–southwest spatial distribution pattern, and ecological space form an east–west spatial distribution pattern. From 1980 to 2020, the standard deviation ellipse area of China’s production space increased, and the growth in the Y-axis direction was significantly higher than that in the X-axis direction, indicating that the production space expanded significantly along the Y-axis direction, namely the northwest to southeast direction. The gravity center of production space shifted 50.931 km to the northwest from 1980 to 2000, and 66.426 km to the northwest from 2000 to 2020 (Table 3). During the study period, the standard deviation ellipse area of living space increased, and the growth in the Y-axis direction was significantly higher than that in the X-axis direction, indicating that the living space expanded significantly along the Y-axis direction, namely the northwest to southeast direction. From 1980 to 2010, the gravity center of living space shifted 55.550 km to the southwest, and 51.639 km to the northwest from 2010 to 2020. During the study period, the standard deviation ellipse area of ecological space was small, and the center of ecological space gravity shifted 25.224 km to southwest China from 1980 to 2020.

### 3.5. Mechanism of Regional Differentiation in China from 2000 to 2020

#### 3.5.1. Detection of Territorial Spatial Regional Differentiation Mechanism

Based on Equation (6), this study explored the mechanism of territorial spatial regional differentiation in the whole country and four types of MFZs. The results showed that the evolution of PLES structure in China was influenced by natural and socio-economic factors. In general, *X*_1_ had the most prominent influence on the formation of PLES, while other influencing factors had significant differences in different regions. Specifically, from a national scale, the impact of *X*_1_ on PLES gradually increased during the study period, and the similar impact of *X*_2_ on PLES also gradually increased. The impact of *X*_3_ on ecological space was greater than that of living space and production space, while the impact of *X*_4_, *X*_5_, and *X*_6_ on ecological space was stronger than that of production space and living space. In major agricultural producing areas, the impact of *X*_2_ on living space was significantly lower than that of production space and ecological space. Similar to the national scale, the impact of *X*_3_ on ecological space was higher than that of living space and production space. The impact of *X*_4_ on living space was stronger than that of production space and ecological space, while the impact of *X*_5_ on living space was lower than that of production space and ecological space. The impact of *X*_6_ on PLES was lower than that of *X*_1_, and the impact of *X*_6_ on production space was lower than that of living space and ecological space.

In the optimized development zone, the impact of each influencing factor on PLES fluctuated greatly in different years. Specifically, the impact of *X*_2_ and *X*_3_ on production space and living space in 2000 was significantly higher than that in 2010 and 2020, while the impact of *X*_2_ and *X*_3_ on ecological space in 2010 and 2020 was significantly higher than that in 2000. The impact of *X*_4_ on ecological space was higher than that of production space and living space, the impact of *X*_5_ on production space was significantly higher than that of living space and ecological space, and the impact of *X*_6_ on living space was the biggest. In key development zone, the impact of *X*_2_ on production space and living space was significantly higher than that of ecological space, and the impact of *X*_3_ on ecological space was significantly higher than that of living space and production space. The impact of *X*_4_ and *X*_5_ on living space was significantly lower than that of production space and ecological space. The impact of *X*_6_ on ecological space was significantly higher than that of living space and production space. In key eco-function zone, the impact of *X*_2_ on PLES increased gradually, while the impact of *X*_3_ on production space and ecological space gradually decreased, and the impact on living space gradually increased. The impact of *X*_4_ and *X*_5_ on production space were higher than those of ecological space and living space, and the impact of X_6_ on ecological space was significantly higher than those of production space and living space.

#### 3.5.2. Evolution Mechanism of Territorial Space

**Figure 9:**
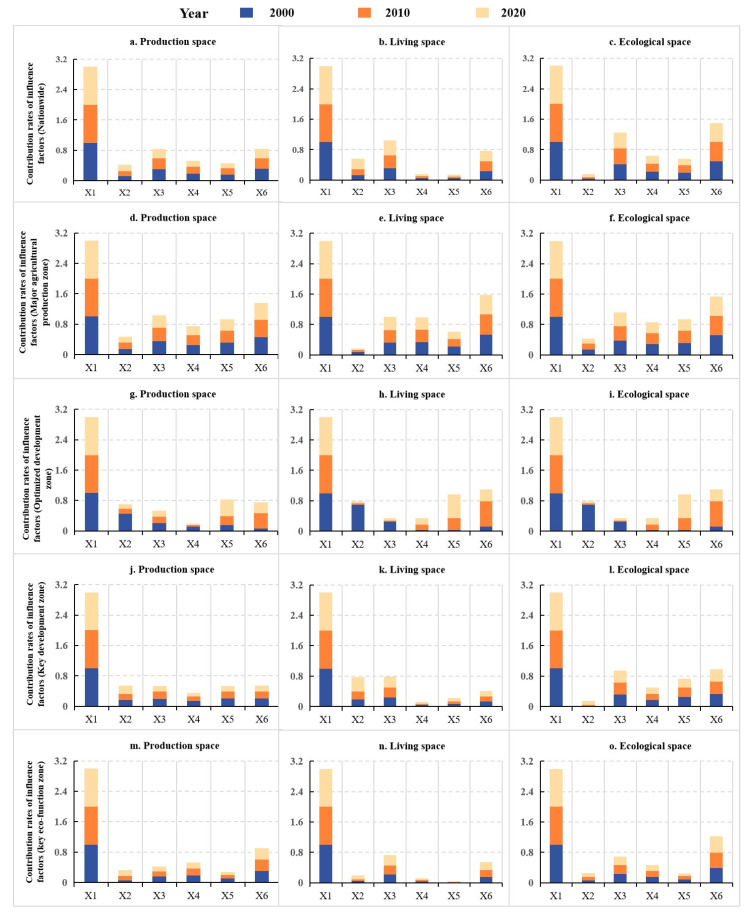
Contribution rates of influence factors from 2000 to 2020. Notes: Variables *X*_1_–*X*_6_ in the figure represent land use intensity, NDVI, population density, average annual temperature, average annual precipitation, and average elevation, respectively.

The results of interactive detection by GeoDetector model showed that the evolution of China’s territorial spatial pattern was formed by the combined effects of natural and socio-economic factors through nonlinear enhancement and dual-factor enhancement, with the nonlinear enhancement the dominant, showing the synergistic enhancement effect. By comparing the interaction factor values of different zones, it could be found that the interaction between *X*_1_ and other factors was significantly stronger than the interaction between other factors (Figure 9). The increase in *X*_1_ would accelerate the evolution of territorial space. Therefore, the interaction degree between *X*_1_ and various factors is the most complex. The evolution of China’s territorial space was influenced by the integration of natural and socio-economic factors. In different regions, there were significant differences in natural environment background, resource and environment carrying capacity, location characteristics, environmental capacity, existing development density, economic structure characteristics, population agglomeration, and participation in international division of labor. However, it could be found that the intensity of interaction between *X*_6_ and other factors in different MFZs was second only to *X*_1_. As an important natural element, X_6_ can effectively limit the range of *X*_2_ and affect the evolution characteristics of territorial spatial structure.

## 4. Discussion

As a big strategic background, the MFZ carries the national will and transmits it to all kinds of planning, and finally guides the layout of PLES through the three-zones and three-lines [4,49,64]. The quantitative relationship and spatial layout of PLES are the application guidance of national strategy at the level of territorial space, and also the real appeal of the public for affluent life, efficient production, and ecological living conditions [5,34,67,68]. Meanwhile, it is also a response to the sustainable development goals of the United Nations [69]. In Europe, land-use functions are classified into three main functions: social, economic, and environmental functions [35]. In China, the classification of PLES is based on the theory of multi-functionality of land use in Europe [34]. For example, Liu et al. scored different land use types according to the primary and secondary functions of the land [44], while Liao et al. established a PLE land classification system for southwestern China [70]. These studies provide the research basis for the classification of PLES in this study.

Based on the remote sensing data of land use monitoring in China and a series of theories and methods of territorial spatial evolution analysis, through the construction of PLES classification system, this study analyzed the evolution patterns of PLES structure of China’s MFZs in the past 40 years. In addition, the GeoDetector model was used to detect the mechanism of territorial spatial regional differentiation of different MFZs. Studies consistently consider that natural and socio-economic factors jointly influence the evolution of territorial spatial structure, most notably human activity [22]. However, the evolution of territorial spatial structure is also affected by national strategic policies and public appeals. Meanwhile, the demarcation of China’s MFZ is based on the differences in economic development level, development intensity, resource and environment carrying capacity, development potential, and development direction within the region. The delineation of optimized development zone, key development zone, restricted development zone, and prohibited development zone will certainly affect the quantity and layout of PLES, and the public demand will also appeal to PLES. Thus, future research needs to further strengthen the research on the impact of national macro strategies and public appeals on the evolution of territorial spatial structure. Besides, it is vital to scientifically explore the evolution process and formation mechanism of PLES in China’s MFZs in the past 40 years, and to effectively connect and provide feedback on the PLES between MFZs, territorial spatial planning, and three-zones and three-lines. Based on the analysis of the evolution process of China’s territorial space over a long period of time and the detection results of regional differentiation mechanism, this study puts forward the following suggestions.

Firstly, the development of the optimized development zone need to focus on improving and upgrading the quality and the transformation of production and living space. However, at present, the living space expansion rate of the optimized development zone in China is the fastest among all MFZs. In the future, it is necessary to further optimize living space, improve supporting functional facilities, and control urban sprawl, and promote more scientific and reasonable layout of living space.

Secondly, key development zone is important carrier to support the country’s future economic development and population agglomeration. This study found that the production space of the key development zone in the last 40 years has decreased, while the increase in living space is not significant. In the future, it is necessary to further improve the urban infrastructure and public services and promote the population and economy to cluster in the urban agglomeration and the core area of the main axis.

Thirdly, the main agricultural producing zone is an important area to guarantee the supply safety of agricultural products in China. The production space of major agricultural producing zone in the past 40 years only increased by 1.22%. In the future, we need to step up efforts to comprehensively improve territorial space and restore the ecological environment, strengthen agricultural infrastructure, improve the distribution and structure of agricultural production, and increase the intensity of development in major agricultural production zone.

Lastly, restricted development zone is an important guarantee for ecological security in China. However, in the past 40 years, the production space and living space in China’s key eco-function zone have increased by 0.98% and 0.22%, respectively, while the ecological space has decreased by 1.20%. In the future, it is necessary to further restrict large-scale and high-intensity industrialization and urbanization in territorial space development and optimize the ecosystem pattern.

## 5. Conclusions

Based on China’s land use data from 1980 to 2020, this study explored the evolution process and the formation mechanism of the PLES structure of China’s MFZs in the past 40 years by combining the theories and methods of territorial spatial pattern evolution, such as territorial spatial transfer matrix, landscape pattern index, standard deviation ellipse, and GeoDetector model. The results are as follows:

(1) During the study period, China’s ecological space was absolutely dominant, and its proportion continued to decrease, while the production space and living space continued to increase. There were significant differences in the proportion of PLES in different types of MFZs.
(2) During the study period, the conversion between land types was frequent, among which the conversion between grassland and other land use spaces was the most frequent. From 1980 to 2000 and 2000 to 2010, the largest conversion was grassland ecological space to other space, and from 2000 to 2010, it was the grassland ecological space to agricultural production space; while from 2010 to 2020, other land use space converted to grassland ecological space was the largest.
(3) During the study period, the COHESION index of ecological space was significantly higher than that of living space and production space, and the COHESION index of rural living space was the lowest. The PD index of agricultural production space and grassland ecological space was high, while the ENN_MN of industrial and mining production space and urban living space was relatively large.
(4) The spatial distribution pattern of production space and living space was northeast to southwest, and the spatial distribution pattern of ecological space was east to west. There was a gradual shift of the PLES to the west during the study period.
(5) The land use intensity had the most prominent influence on the formation of PLES, and the intensity of other influencing factors varied significantly in different regions. The evolution of China’s territorial spatial pattern was a synergistic enhancement effect of natural factors and socio-economic factors through nonlinear enhancement and dual-factor enhancement.

It is expected that the results of this study and the proposed policy recommendations can provide scientific support for the optimal management and high-quality development of territorial space in China and other regions with similar dominant functions.

## Figures and Tables

**Table 1:** Land use types based on dominant function.

Land Use Classification Based on Dominant Function and Production-Living-Ecological Land Types		National Land Use Classification System
	First-Level Type	Second-Level Type
Production space	Agricultural production space	Paddy field, dry land
	Industrial and mining production space	Mining and transportation land
Ecological space	Forestland ecological space	Forestland, shrub area, wood land, other forest land
	Grassland ecological space	High coverage grassland, medium coverage grassland, low coverage grassland
	Water ecological space	River and canals
		Lakes
		Reservoir, pit, and ponds
		bottom land
	Other ecological space	Swampland, bare soil
		Bare rock
Living space	Urban living space	Urban land
	Rural living space	Rural residential land

**Table 2:** Interaction types of Geodetector model.

Criterion	Interaction
*q*(*X*_1_∩*X*_2_) < min[*q*(*X*_1_), *q*(*X*_2_)]	The interaction of *X*_1_ and *X*_2_ factors weakens the nonlinearity
min[*q*(*X*_1_), *q*(*X*_2_)] < *q*(*X*_1_∩*X*_2_) < max[*q*(*X*_1_), *q*(*X*_2_)]	The interaction of *X*_1_ and *X*_2_ factors weakens the single-factor nonlinearity
*q*(*X*_1_∩*X*_2_) > max[*q*(*X*_1_), *q*(*X*_2_)]	The interaction of *X*_1_ and *X*_2_ factors enhances the dual-factor
*q(X*_1_∩*X*_2_) = *q*(*X*_1_) + *q*(*X*_2_)	The *X*_1_ and *X*_2_ factors are independent
*q*(*X*_1_∩*X*_2_) > *q*(*X*_1_) + *q*(*X*_2_)	The interaction of *X*_1_ and *X*_2_ factors enhances the nonlinearity

**Table 3:** Standard deviation ellipse parameter of PLES pattern during 1980–2020.

Year	Ecological Space				Production Space				Living Space			
	Latitude and longitude of Central Point	Major Axis/km	Minor Axis/km	Azimuth Angle	Major Axis/km	Minor Axis/km	Azimuth Angle	Major Axis/km	Major Axis/km	Minor Axis/km	Azimuth Angle	Major Axis/km
1980	35.20° N; 113.39° E	1267.67	894.70	36.41	35.81° N 115.58° E	1091.73	824.22	23.55	36.82° N 100.99° E	1583.86	1144.9	85.95
1990	35.31° N; 113.48° E	1280.55	892.98	36.49	35.86° N 115.55° E	1103.07	852.33	23.65	36.80° N 100.94° E	1571.62	1142.22	86.57
2000	35.59° N; 113.64° E	1309.96	896.53	36.61	35.65° N 115.49° E	1095.45	845.51	21.39	36.74° N 100.83° E	1569.92	1142.87	87.48
2010	35.700° N; 113.49° E	1315.49	934.95	38.04	35.32° N 115.49° E	1086.94	844.13	18.80	36.72° N 100.85° E	1568.43	1141.37	87.38
2020	35.85 N; 113.10 E	1341.54	1012.80	41.90	35.44° N 115.29° E	1062.34	893.53	23.51	36.69° N 100.81° E	1591.54	1142.16	88.04

## Data Availability

The data presented in this study are available on request from the corresponding author.
